# Infrared actuation-induced simultaneous reconfiguration of surface color and morphology for soft robotics

**DOI:** 10.1038/s41598-017-17904-y

**Published:** 2017-12-13

**Authors:** Seyedali Banisadr, Jian Chen

**Affiliations:** Department of Chemistry and Biochemistry, University of Wisconsin-Milwaukee, Milwaukee, 3210, North Cramer Street, Milwaukee, WI 53211 United States

## Abstract

Cephalopods, such as cuttlefish, demonstrate remarkable adaptability to the coloration and texture of their surroundings by modulating their skin color and surface morphology simultaneously, for the purpose of adaptive camouflage and signal communication. Inspired by this unique feature of cuttlefish skins, we present a general approach to remote-controlled, smart films that undergo simultaneous changes of surface color and morphology upon infrared (IR) actuation. The smart film has a reconfigurable laminated structure that comprises an IR-responsive nanocomposite actuator layer and a mechanochromic elastomeric photonic crystal layer. Upon global or localized IR irradiation, the actuator layer exhibits fast, large, and reversible strain in the irradiated region, which causes a synergistically coupled change in the shape of the laminated film and color of the mechanochromic elastomeric photonic crystal layer in the same region. Bending and twisting deformations can be created under IR irradiation, through modulating the strain direction in the actuator layer of the laminated film. Furthermore, the laminated film has been used in a remote-controlled inchworm walker that can directly couple a color-changing skin with the robotic movements. Such remote-controlled, smart films may open up new application possibilities in soft robotics and wearable devices.

## Introduction

Cephalopods, such as cuttlefish, have extraordinary capabilities to instantaneously, simultaneously, and reversibly change their skin color, pattern, and texture in response to environmental stimuli. They use such dynamic modulation of their body appearance for adaptive camouflage and signal communication^1–3^. The coloration in their skins is due to the chromatophores embedded in sacs which are controlled by multiple radial muscles, as well as the structural colors (iridophores, and leucophores). Cuttlefish also can rapidly change the surface morphology of their skins from smooth to spiky, which is unique in the animal kingdom. Cephalopods, including cuttlefish, commonly swim in large schools. It is clear that the coordination of the movements of members in a school plays a crucial role for preserving the integrity of the school. Iridophores of their skins provide them with an exceptional means of communication^3^. One distinct optical feature of the iridophores is the dependence of the peak wavelength of the reflected light on the angle of observation/incidence, which is used by members of a school for coordination of their swimming direction. Moreover, the polarized light reflected from the iridophores enable them to communicate with the other members of the group in a hidden way, since cephalopods are especially sensitive to polarized light^3^. These unique features of their skins have attracted growing interest in recent years to develop soft material systems and devices to mimic such functions for potential applications in soft robotics and wearable devices^4–14^.

Significant progress has been made recently in engineering some of the key functions inspired by cuttlefish skins^4–11^. For instance, Morin and coworkers utilized soft robots equipped with microfluidic networks to exhibit active camouflage and displays by pneumatic pumping of chemical dye fluids through the network^4^. Yu and coworkers developed adaptive optoelectronic camouflage systems that can autonomously sense and adapt to the coloration of their surroundings^5^. Wang and coworkers demonstrated an electro-mechano-chemically responsive elastomer system that can produce voltage-controlled on-demand fluorescent patterns^6^. The fluorescent signals result from large deformation of a stretchable elastomer covalently coupled with spiropyran-based mechanochromic molecules under the control of electric fields. More recently, Larson and coworkers reported a highly stretchable electroluminescent material composed of a ZnS phosphor-doped dielectric elastomer layer sandwiched between layers of hydrogel electrodes, which can change illuminance and capacitance under voltage-induced deformation^9^.

The colorations in the aforementioned studies were based on chemical dyes and phosphors. In contrast, structural colorations are widely found in nature, including cephalopods, where colors originate from micro- or nanostructures^15,16^. One main advantage of structural colors is that they are not easily degraded by environmental conditions such as ultraviolet (UV) light, heat, oxygen, and moisture. An important source of structural colorations is from photonic crystals, which are 1-D, 2-D, or 3-D ordered nanostructures of two or more media with different refractive indices arranged in a spatially periodic fashion. Due to the periodic arrangement of materials with different refractive indices, a photonic bandgap appears, which leads to selective prohibition of the light of certain wavelength from propagating through the photonic crystal. A 3D photonic crystal diffracts light of a specific wavelength as determined by Bragg’s law (Equation 1):

1 $${m}\lambda =2nd\,\sin \,\theta $$

Where *m* is the order of diffraction, *λ* is the diffracted wavelength of the incident light, *n* is the effective refractive index of the system, *d* is the spacing between the diffracting planes, and *θ* is the Bragg glancing angle between the incident light and diffracting planes^17^. There has been great interest in responsive photonic crystals, which can change the photonic bandgaps upon exposure to external stimuli such as heat, chemicals, mechanical strains, light, electric fields, and magnetic fields^15–19^.

A promising new strategy towards cuttlefish-inspired smart films is to integrate an elastomeric photonic crystal^20–25^ with a mechanical actuator^26–29^, where the actuator provides mechanical strains that can induce the out-of-plane deformation and change the photonic bandgap of the elastomeric photonic crystal. Liquid crystalline elastomers (LCEs) are excellent candidates for mechanical actuators because they can translate small molecular movements triggered by an external stimulus such as heat or light into large, fast, and reversible mechanical motions^30–38^. However, this new strategy for bio-inspired smart films has been underexplored so far in the literature. Recently, Guo, Wei, and coworkers reported thermoresponsive photonic crystal composite actuators, where SiO_2_ photonic crystals were embedded in the matrix of excessive LCE or liquid crystalline polymer networks^39,40^. Upon exposure to heat using an external heating source, the photonic crystal composite actuators could change the shape as well as the color. However, the need of an external heating source renders these smart films impractical for soft robotics applications.

Light-driven actuation is highly desirable for various applications, because it not only offers remote, spatial, and temporal control over the actuators, but also permits sophisticated control over light direction, wavelength, intensity, and polarization^28,29^. Hence the light allows for complicated actuation movements without using additional energy sources and complex components, which can considerably simplify the design of actuator devices and reduce their sizes and weights. Infrared (IR) light is usually better than either UV or visible light for light-driven actuation, because IR light can penetrate much deeper in most polymeric materials^31,41^ and it generally causes little material damage compared with UV or visible light. In addition, numerous near IR absorbing materials and IR lasers with different wavelengths are available for different target applications.

We have previously demonstrated that the IR light-driven LCE composite films based on near IR absorbing fillers, such as single-wall carbon nanotubes (SWNTs) and near IR (NIR) dyes, are suitable candidates for soft robotics applications^42,43^. In the present study, we report a general approach to remote-controlled, smart films that undergo simultaneous changes of surface color and morphology upon IR actuation. The smart film has a reconfigurable laminated structure that comprises an IR-responsive nanocomposite actuator layer and a mechanochromic elastomeric photonic crystal layer, which can be disassembled with appropriate tools and reassembled based on various needs. Upon global or localized IR irradiation, the nanocomposite actuator layer exhibits fast, large, and reversible strain in the irradiated region, which causes a synergistically coupled out-of-plane deformation of the laminated film and structural color change in the mechanochromic elastomer layer in the same region.

Our IR-actuated laminated film design has brought together several important features: i) Large, fast, and reversible remote-controlled actuation; ii) Intrinsically coupled change of both surface color and morphology; iii) IR laser-induced localized actuation; iv) Reconfigurability of the laminated film through disassembly with appropriate tools and reassembly.

## Results and Discussion

### The Laminated Films

**Figure 1:**
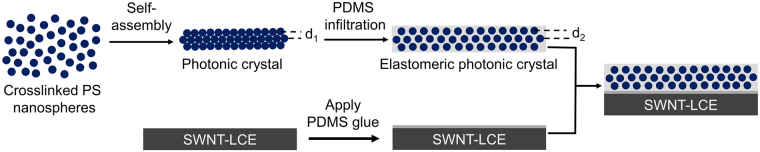
Schematic illustration of the fabrication of the laminated film.

**Figure 2:**
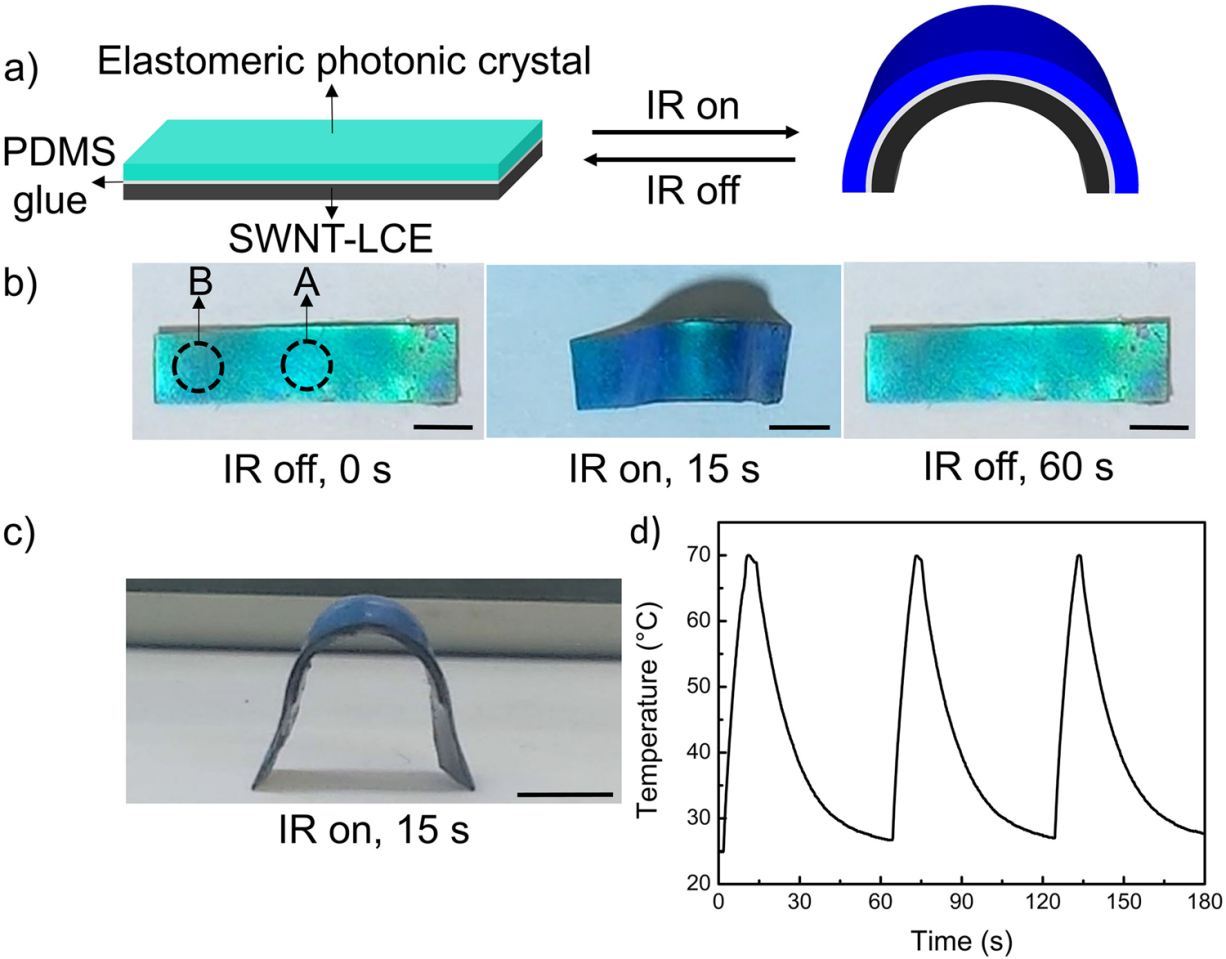
(**a**) Scheme of the laminated film undergoing bending towards the SWNT-LCE side upon IR irradiation. (**b**) Photographs (top view) of reversible bending and unbending of the laminated film in response to global IR irradiation. (**c**) Photograph (side view) of bent laminated film upon global IR irradiation. (**d**) Temperature of the laminated film as a function of on and off cycles of global IR irradiation. Scale bar: 5 mm.

The fabrication procedure for the laminated films is illustrated in Fig. 1 and the details are described in the Methods section. A schematic diagram of the laminated film is further illustrated in Fig. 2a. The typical thickness of the 0.1 wt% SWNT-LCE film and elastomeric photonic crystal film used in this study is around 252 μm and 23 μm, respectively. Compared with the 0.1 wt% SWNT-LCE film, the laminated film exhibits roughly comparable Young’s modulus and tensile strength but nearly 40% increase in elongation at break (Table S1). In the laminated films, good adhesion between the actuator and structural color layers is crucial for the reversible and steady actuation. Herein, the PDMS silicone resin with a thickness of ~15 µm has been employed as the adhesive interlayer to supply sufficient bond strength for restricting the negative in-plane strain-induced contraction of the SWNT-LCE layer. We have never observed any delamination of well-cured laminated films during IR actuation experiments. One additional advantage of using the PDMS silicone glue is that it allows for the disassembly of the laminated film with appropriate tools. We have found that the SWNT-LCE layer can be peeled off intact with tweezers. There is no visible silicone glue residue left on the SWNT-LCE film. Moreover, most of the silicone glue residue left on the elastomeric photonic crystal film can be readily removed using a razor. The facile disassembly of the laminated films with appropriate tools not only allows for full recovery of SWNT-LCE and elastomeric photonic crystal films, but also enables the reconfiguration of laminated films by, for example, recoupling the SWNT-LCE film with another elastomeric photonic crystal film that has different color, or pairing the elastomeric photonic crystal film with another SWNT-LCE film that has different nematic director orientation.

### IR Actuation of the Laminated Films

Upon global IR irradiation using a Torch NIR light source, well-dispersed SWNTs can efficiently absorb and transform IR light into thermal energy, thereby serving as numerous nanoscale heaters uniformly embedded in the LCE matrix. The absorbed thermal energy, if sufficient, then induces the LCE N–I phase transition^42,43^. As the result, the SWNT-LCE layer in the laminated film undergoes a significant in-plane negative strain. Due to the PDMS glue-mediated strong mechanical coupling of the SWNT-LCE layer with the elastomeric photonic crystal layer, the in-plane contraction of the SWNT-LCE layer is impossible and instead the in-plane negative strain bends the elastomeric photonic crystal layer towards the SWNT-LCE side (Fig. 2a–c). The IR-induced bending actuation of the laminated film then causes a simultaneous color change of the elastomeric photonic crystal film from cyan to blue (Fig. 2b; Movie S1). Figure 2c shows the side-view image of the IR-induced bent laminated film, which indicates the curvature of the film reaches 0.28 mm^−1^ only after 15 seconds of global IR irradiation. The IR-induced bending curvature of the laminated film can be controlled by tuning the IR light intensity or the film thickness^43^. As shown in Fig. 2d, the temperature of the laminated film reaches about 70 °C soon after the IR light is turned on, which is well above the N-I phase transition temperature of the SWNT-LCE layer (~64.6 °C). The maximum temperature of the LCE layer can be tuned by adjusting the loading level of SWNTs or IR light intensity^42,43^.

**Figure 3:**
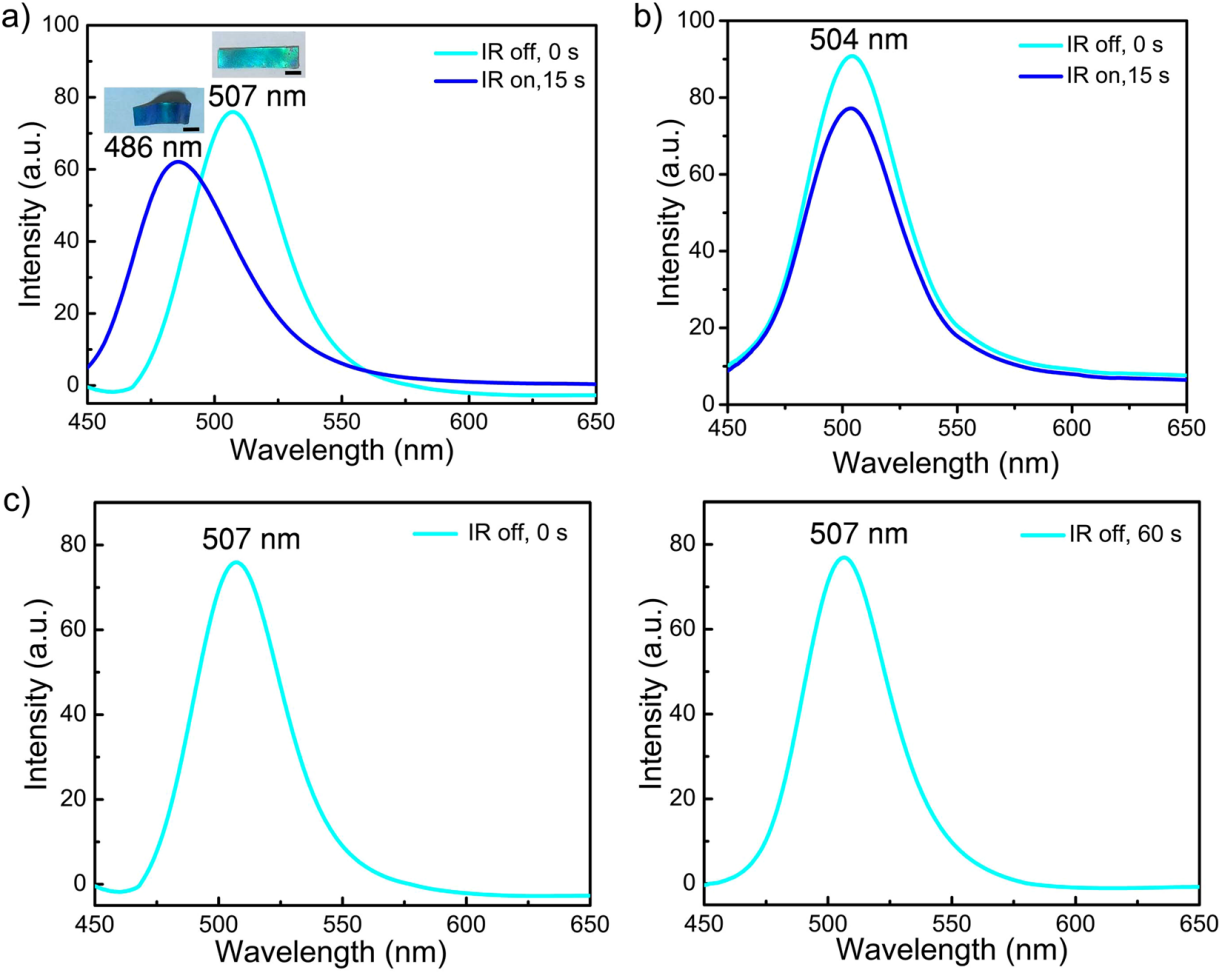
(**a**) Reflection spectra (*θ* = 90°) of the highly deformed region of the laminated film, represented by the encircled area A in Fig. 2b, before and upon global IR irradiation, respectively. Inset images are corresponding photographs. (**b**) Reflection spectra (*θ* = 90°) of the less deformed region of the laminated film, represented by the encircled area B in Fig. 2b, before and upon global IR irradiation, respectively. (**c**) Reflection spectra (*θ* = 90°) of the unbent laminated film before and after global IR irradiation. Scale bar: 5 mm.

The observed color change of the laminated film is due to a combination of a decrease in lattice constant *d* of the elastomeric photonic crystal layer in the highly deformed region, represented by encircled area A, and a change of viewing angle *θ* in the less deformed regions, represented by encircled area B (Fig. 2b). Upon IR-induced bending of the laminated film, the photonic bandgap of highly deformed area A of the mechanochromic layer undergoes a blueshift from 507 nm to 486 nm (*θ* = 90°) due to a decrease of *d* in the vertical direction, while the reflectance intensity in the same area only decreases slightly (Fig. 3a). Since the laminated film is elastomeric by nature, it can rapidly revert to its original shape and color upon turning off the IR light (Fig. 3c). In contrast, the photonic bandgap of area B remains unchanged at 504 nm (*θ* = 90°) upon IR irradiation (Fig. 3b).

**Figure 4:**
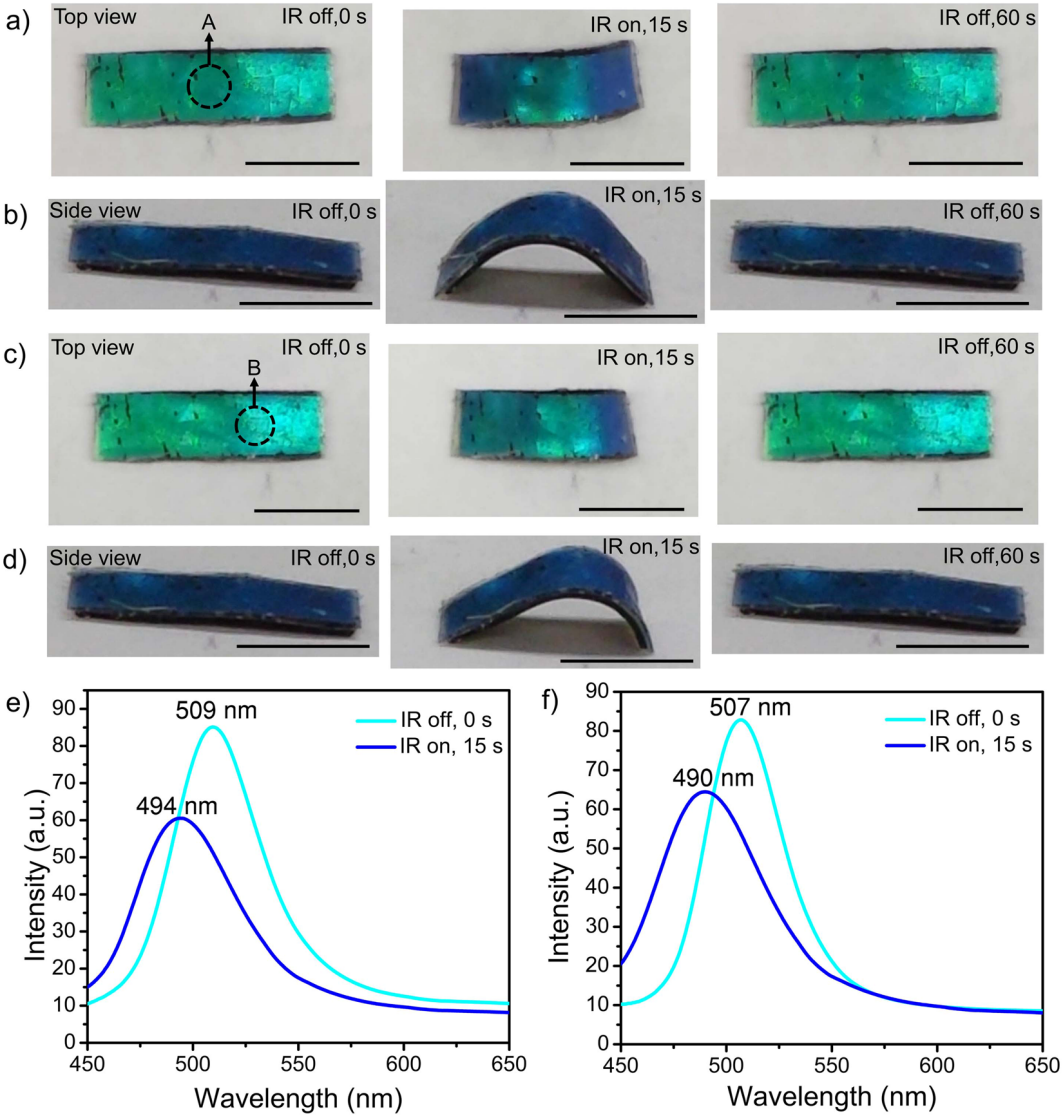
Photographs of localized IR actuation in the encircled area A of the laminated film at (**a**) top view and (**b**) side view, respectively. Photographs of localized IR actuation in the encircled area B of the laminated film at (**c**) top view and (**d**) side view, respectively. Scale bar: 5 mm. (**e**) Reflection spectra (*θ* = 90°) of the encircled area A (Fig. 4a) of the laminated film before and upon localized IR irradiation, respectively. (**f**) Reflection spectra (*θ* = 90°) of the encircled area B (Fig. 4c) of the laminated film before and upon localized IR irradiation, respectively.

To demonstrate the feasibility of localized bending actuation, the 808 nm IR laser was pointed to a selected region such as area A or B one at a time (Fig. 4). As a result of localized heating, the laminated film can exhibit coupled bending and color change at a desired region. Similar to global IR actuation (Fig. 3), the photonic bandgap of highly deformed region in the laminated film undergoes a blueshift of 15–17 nm (*θ* = 90°) in localized IR actuation (Fig. 4e,f).

**Figure 5:**
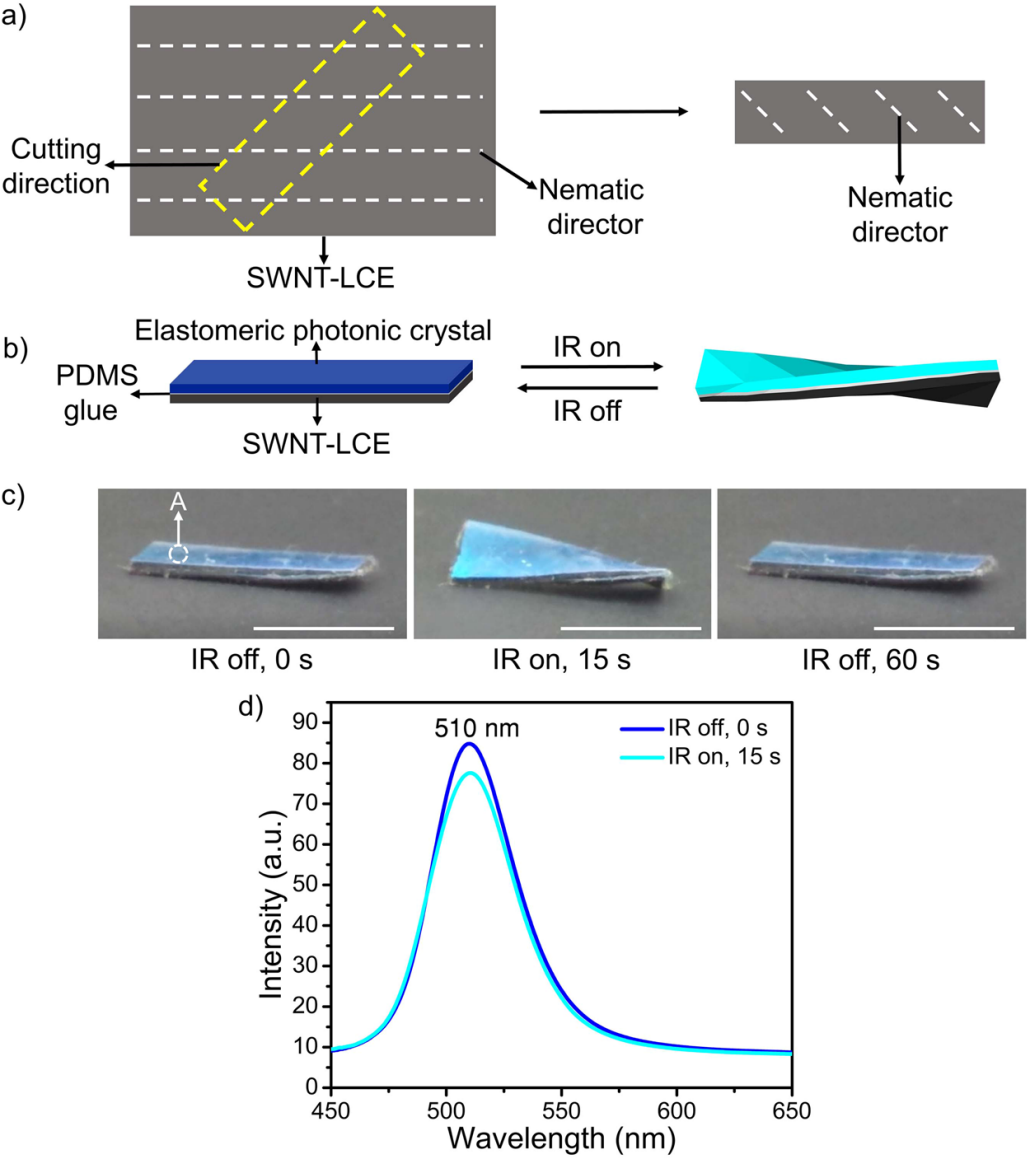
(**a**) Schematic illustration of the SWNT-LCE film subjected to a cutting angle of 45° relative to the nematic director. (**b**) Scheme of the laminated film undergoing twisting upon IR irradiation. (**c**) Photographs (side view) of reversible twisting and untwisting of the laminated film in response to global IR irradiation. Scale bar: 5 mm. (**d**) Reflection spectra (*θ* = 90°) of the encircled area A (Fig. 5c) of the laminated film before and upon global IR irradiation, respectively. In order to acquire the reflection spectra (*θ* = 90°) of the laminated film, the fiber optic probe is oriented perpendicular to the plane of the area A of the laminated film for both untwisted and twisted shapes.

In aforementioned laminated films, both the mesogenic units and IR-induced negative strain are oriented along the long axis of the SWNT-LCE film, which leads to bending actuation. In contrast, a twisted actuation can be achieved by cutting the SWNT-LCE nanocomposite film at 45° angle relative to the nematic director (i.e. the hot-drawing direction) (Fig. 5a). In response to global IR irradiation, the generated negative strain is 45° relative to the long axis of the SWNT-LCE film, which produces the twisting deformation of the laminated film when its temperature reaches above the N-I phase transition temperature (Fig. 5b,c). This twisting deformation causes a change in the side-view reflection color of the laminated film from blue to cyan, which is mainly attributed to the change of viewing angle. As shown in Fig. 5d, the photonic bandgap of area A remains unchanged at 510 nm (*θ* = 90°) upon global IR irradiation. Like the bending deformation, the twisted laminated film returns to its original shape upon turning off the IR light (Fig. 5c).

**Figure 6:**
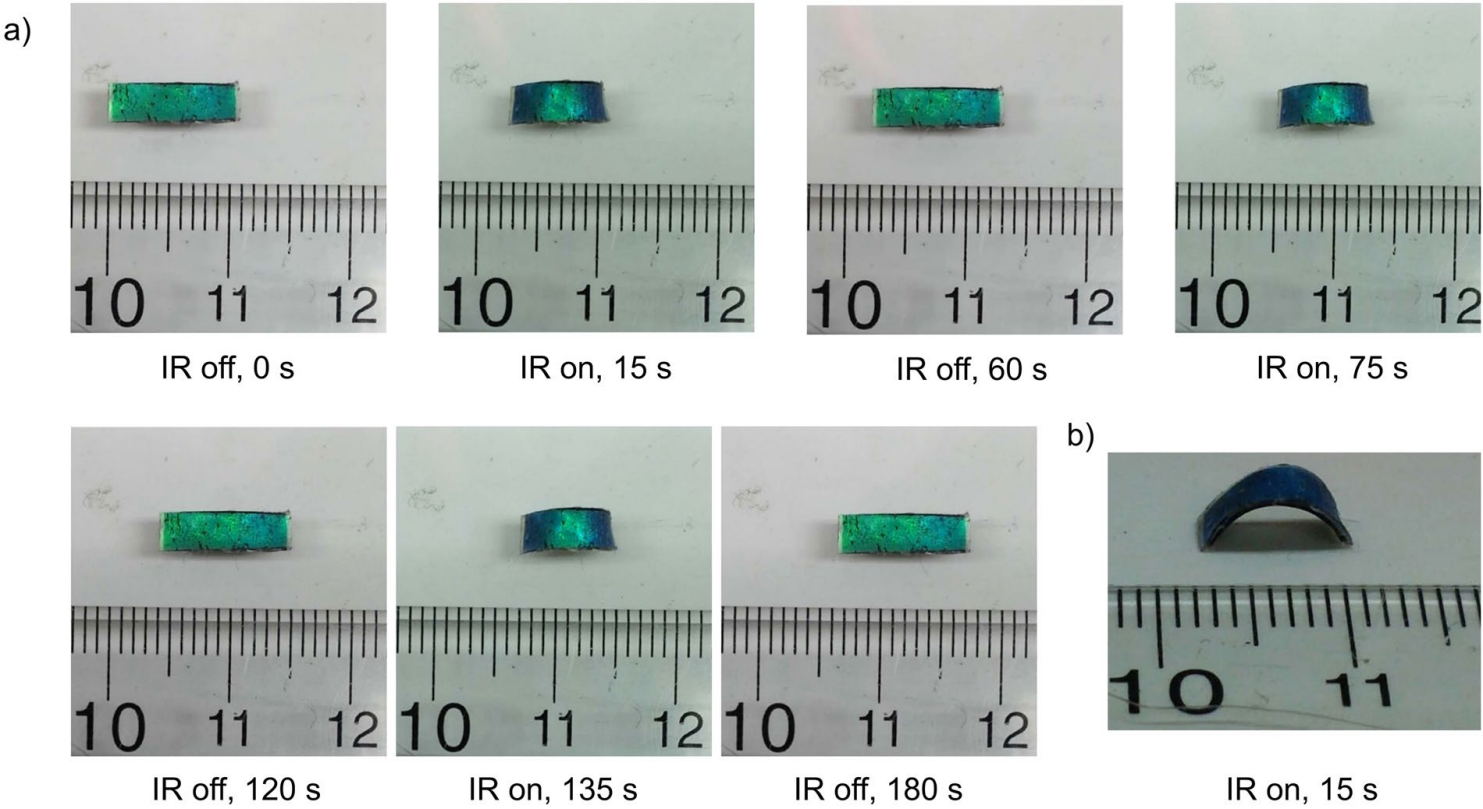
(**a**) Photographs (top view) of the laminated film-based inchworm walker movements on glass in response to on and off cycles of global IR irradiation. (**b**) Photograph (side view) of the laminated film-based inchworm walker movement from left to right on paper in response to global IR irradiation during the first on and off cycle.

Despite recent growing interests in bio-inspired soft robotics such as inchworm walkers^43,44^, soft robotic devices that can exhibit synergistic coupling of color change and out-of-plane deformation in motion are still very rare and require air pressure tubings^8,9^. In this study, we show that the laminated film-based inchworm walkers are capable of IR-induced simultaneous reconfiguration of surface color and morphology during the movement. As seen in Fig. 6a, the laminated film moves on glass from left to right like an inchworm in response to on and off cycles of Torch NIR light (Movie S2). In the beginning of each actuation cycle (i.e. IR is turned on), the front part of the film forms the stationary point on glass while the back part of the film slides forward. In the end of each actuation cycle (i.e. IR is turned off), the back part of the film forms the stationary point on glass while the front part of the film pushes forward. The directional inchworm-like movements of the laminated film are due to the asymmetric bending of the film under global IR irradiation, which is evident from close inspection of the side view image of the bent film (Fig. 6b). The front part (Thickness: ~290 μm) of the laminated film is slightly thinner than the back part (Thickness: ~302 μm). As a result, the front part of the film bends more than the back part upon global IR irradiation (Fig. 6b). We have previously found that the bending curvature of a bilayer film decreases with increase in film thickness^43^. We have also discovered that the same laminated film changes the moving direction to the opposite when it is turned 180° horizontally along the long axis. This observation further confirms that the thinner end of the film always serves as the front moving end during the IR actuation cycles.

The friction between the laminated film and substrate is crucial for inchworm walker’s movements. We have found that the same laminated film moves much faster with more constant speed on glass (0.036 mms^−1^) than on paper (0.022 mms^−1^) under same global IR actuation conditions. Since the paper substrate provides lower friction for the laminated film than the glass substrate, it is difficult for one end of the asymmetrically bent laminated film to form an effective stationary point on paper during some actuation cycles. Therefore the moving speed of the laminated film on paper is considerably lower and fluctuating compared with that on glass, while the moving direction remains the same as on glass due to the formation of similar asymmetric bending upon global IR irradiation (Fig. 6b). The walking velocity of the inchworm walker could be significantly enhanced by further optimization of the thicknesses at front and back parts of the laminated film, and friction between the film and substrate. The fact that the inchworm walker exhibits reversible and coupled reconfiguration of color and shape for repeated actuation cycles confirms the flexibility and durability of the laminated films, which are highly desirable for soft robotics applications. We have conducted over 60 IR actuation cycles for each laminated film and ~250 IR actuation cycles for all laminated films in total, and we have never observed any delamination of the laminated films during these experiments.

## Conclusion

In conclusion, we have developed a bio-inspired general approach to remote-controlled, smart films that undergo simultaneous changes of surface color and morphology in the irradiated region upon global or localized IR actuation. Our approach is based on a laminated structure that directly couples an IR-responsive nanocomposite actuator layer with a mechanochromic elastomeric photonic crystal layer. The facile disassembly of the laminated films with appropriate tools not only allows for full recovery of actuator and mechanochromic films, but also enables the reconfiguration of laminated films by reassembly of different layers. We have shown that the laminated films can be incorporated into soft robotic devices such as inchworm walkers, which are capable of remote-controlled coupled reconfiguration of surface color and morphology during the movement. The flexibility and durability of the laminated films are essential for repeated IR actuation cycles that enable soft robotic motion. Although we have focused on using SWNT-LCE nanocomposites as IR-responsive actuators and elastomeric photonic crystals as mechanochromic materials in this study, our approach can be easily extended to other types of IR-absorbing fillers, thermo-responsive shape-changing polymers, and mechanochromic elastomers. Such IR-actuated, reconfigurable films could enable new functions in soft robots and wearable devices.

## Methods

### Preparation and Characterization of 0.1 wt% SWNT-LCE Nanocomposite Films

The preparation of 0.1 wt% SWNT-LCE nanocomposite films was based on our previous report^43^. The resulting SWNT-LCE films were annealed at 40 °C for 2 h to reach an equilibrated state. The stretching ratio of the annealed films for this study was approximately 150%. The stretching ratio of the film is defined by Equation 2.

2 $$Stretching\,Ratio=(L/{L}_{o})({100}{ \% })$$

Where *L*_*o*_ is the initial length of the film and *L* is the final length of the film after annealing. The intensity of UV irradiation for photopolymerization was measured using a Newport power meter (model 1918-C) with a UV detector (918D-UV-OD3). The nematic-isotropic (N-I) phase transition temperature of a representative 0.1 wt% SWNT-LCE film is ~64.6 °C, which was acquired using a TA Instrument differential scanning calorimeter Q10 under Ar^43^. The film thickness was measured with a Mitutoyo Digital Micrometer. Scanning electron microscopy (SEM) was performed using a Hitachi S-4800 field emission scanning electron microscope. Chemical structures of the monomers, crosslinker, and UV initiator that were used to synthesize the side-on LCE matrix are shown in Fig. S1a ^45^. The excellent dispersion of SWNTs in the LCE matrix was confirmed by photography (Fig. S1b) and SEM (Fig. S1c). In addition, SEM shows that SWNTs are partially orientated along the hot-drawing direction. The diameters of SWNTs shown in Fig. S1c are significantly inflated because the SEM image contrast stems from local potential differences between conductive nanotubes and insulating polymer matrix^42^.

### Preparation and Characterization of Crosslinked Polystyrene (PS) Nanospheres

Monodisperse crosslinked PS nanospheres were synthesized by emulsion polymerization, according to a modified literature procedure^46^. Initially, styrene monomer (10.0 g) was magnetically stirred with a mixture of divinylbenzene (0.50 g), sodium dodecylbenzenesulfonate (0.084 g) in 120 mL of deionized water at 300 rpm for 15 min in a 250 mL three-necked flask equipped with a reflux condenser. Subsequently, the reaction flask was purged by nitrogen bubbling at room temperature for 15 min, followed by increasing the reaction temperature to 80 °C using a heating oil bath. After keeping the reaction temperature stable for 45 min, potassium persulfate (0.10 g) was introduced into the reaction mixture. The polymerization was terminated after 5 h, followed by cooling down to room temperature. Finally, the residual styrene and sodium dodecylbenzenesulfonate were removed by repeated cycles of washing, centrifugation, and redispersing in deionized water.

The average diameter (*D*_*n*_) and the coefficient of variation (*C*_*v*_) of the particles were determined from SEM observations of 100 particles to ensure the accuracy of measurements. The *D*_*n*_ and *C*_*v*_ of synthesized PS particles are approximately 180 nm and 3.5%, respectively.

### Preparation and Characterization of Elastomeric Photonic Crystal Films

The elastomeric photonic films were prepared according to a modified literature method^21^. First, a thin film of PDMS precursors (base to curing agent ratio = 10:1) was spin-coated on a clean glass substrate, followed by curing at 80 °C for 4 h. Subsequently, the surface of PDMS thin film was rendered hydrophilic by treating with oxygen plasma for 1 min (Zepto, Diener Electronic). To obtain a well-ordered close-packed crystal structure of the PS nanospheres on the plasma-treated PDMS substrate, the silicone oil-covering self-assembly technique was employed. After self-assembly, the silicone oil was carefully removed using Kim wipe, followed by rinsing the film with isopropyl alcohol. Subsequently, the interstitial voids among the PS particles were infiltrated with the PDMS precursors diluted with hexamethyldisiloxane to reduce the viscosity. The ratio of base:curing agent:hexamethyl disiloxane is 10:1:10. The PDMS precursors were then partially cured at room temperature overnight, followed by removing the excess PDMS precursors, and then fully cured at 70 °C for 3 h. The resulting elastomeric photonic crystal film was then carefully peeled off the substrate using a razor.

Both PS photonic crystal assemblies and corresponding elastomeric photonic crystal films were characterized by SEM and reflection spectroscopy. The reflection spectra were acquired using a fiber optic VIS-NIR spectrometer (USB2000, Ocean Optics). The 180 nm particles formed close-packed photonic crystal assembly (Fig. S2a), which has a bright blue structural color under normal light incidence (*θ* = 90°) (inset photograph of Fig. S2b). As shown in Fig. S2b, the photonic bandgap corresponding to (111)-crystalline planes generates a Bragg reflection peak at *λ* = 429 nm.

The elastomeric photonic crystal film was produced by infiltrating the aforementioned PS photonic crystal assembly with diluted PDMS precursors followed by curing, which leads to a change in the structural color of the film from blue to cyan (inset photograph of Fig. S2d). SEM reveals that the interstitial voids among the PS nanospheres are filled with PDMS (Fig. S2c). Reflection spectroscopy (*θ* = 90°) confirms that the PDMS infiltration results in a redshift in the photonic bandgap from 429 nm to 507 nm (Fig. S2d), which is due to an increase in the lattice constant *d*, as defined in Fig. 1, from *d*_1_ = 149 nm in the PS photonic crystal assembly to *d*_2_ = 164 nm in the elastomeric photonic crystal film.

As with other 3D photonic crystals, our elastomeric photonic crystal film exhibits the iridescent reflection color that depends on the viewing angle. The top view, and side view photographs of the elastomeric photonic crystal film are shown in Fig S3a and S3b, respectively. Figure S3c and S3d show that the structural color of the elastomeric photonic crystal film changes from cyan to blue upon in-plane and out-of-plane mechanical deformations, respectively.

### Preparation and IR Actuation of the Laminated Films

The preparation procedure for the laminated films is schematically illustrated in Fig. 1. First, a thin layer of PDMS precursors (base:curing agent mixing ratio = 10:1) was applied to a 0.1 wt% SWNT-LCE nanocomposite film as glue, and the resulting film was then placed onto the elastomeric photonic crystal film. The silicone glue was allowed to fully cure at room temperature for 48 h. The thickness of silicone glue layer was measured by digital optical microscopy. Mechanical properties of the 0.1 wt% SWNT-LCE and laminated films were characterized using a Shimadzu Autograph AGS‐J universal tester with a 500 N cell load and pneumatic side‐action grips. Tensile tests were carried out along the strain direction of SWNT-LCE films at a strain rate of 0.5 mm/min at room temperature.

For global IR actuation, where the whole film was exposed to IR irradiation, the Torch flashlight (Wicked Lasers), which provides >90% of the light in the NIR region, was used as a NIR light source at a light intensity of 11 mW mm^−2^. For localized IR actuation, where only selected film region was exposed to IR irradiation, the 808 nm IR laser was used as a NIR light source at a light intensity of 45 mW mm^−2^. The light intensities of NIR light sources were measured using a Newport power meter (model 1918-C) with an IR detector (918D-IR-OD3). Temperatures of the laminated films during IR actuation experiments were measured with a non-contact infrared thermometer (MICRO-EPSILON thermoMETER LS), which was found to be in good agreement (within ± 2 °C) with a traditional thermometer.

### Data availability

The data of this study are included in this published article and its Supplementary Information files.

## Electronic supplementary material

Supplementary Information

Movie S1

Movie S2
