# Supplementary material for: Infrared actuation-induced simultaneous reconfiguration of surface color and morphology for soft robotics
Source: Sci Rep. 2017 Dec 13;7:17521. doi: 10.1038/s41598-017-17904-y (PMC5727498; doi:10.1038/s41598-017-17904-y)
Supplement: Supplementary file 1 — Supplementary Information [file 41598_2017_17904_MOESM1_ESM.pdf]

Supplementary information

Infrared actuation-induced simultaneous reconfiguration of surface color and morphology for soft robotics

Seyedali Banisadr & Jian Chen*

Department of Chemistry and Biochemistry, University of Wisconsin-Milwaukee, Milwaukee, 3210 North Cramer Street, Milwaukee, WI 53211, United States

Correspondence and requests for materials should be addressed to J.C. (email: jianchen@uwm.edu)

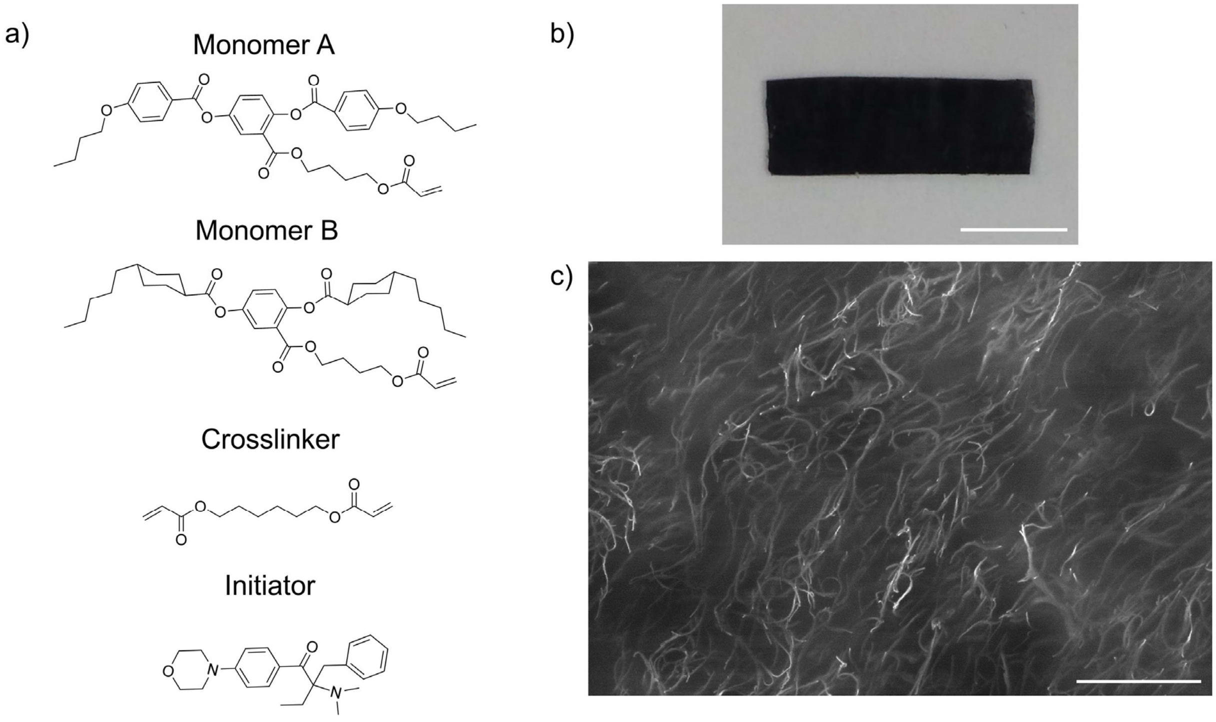

Figure S1. (**a**) Chemical structures of monomers, crosslinker, and initiator used in the preparation of LCE composites. (**b**) Photograph of the 0.1 wt% SWNT-LCE film. Scale bar: 5 mm. (**c**) SEM image of SWNTs in the 0.1 wt% SWNT-LCE film. The hot-drawing direction is roughly 50° relative to the scale bar. Scale bar: 5 µm.

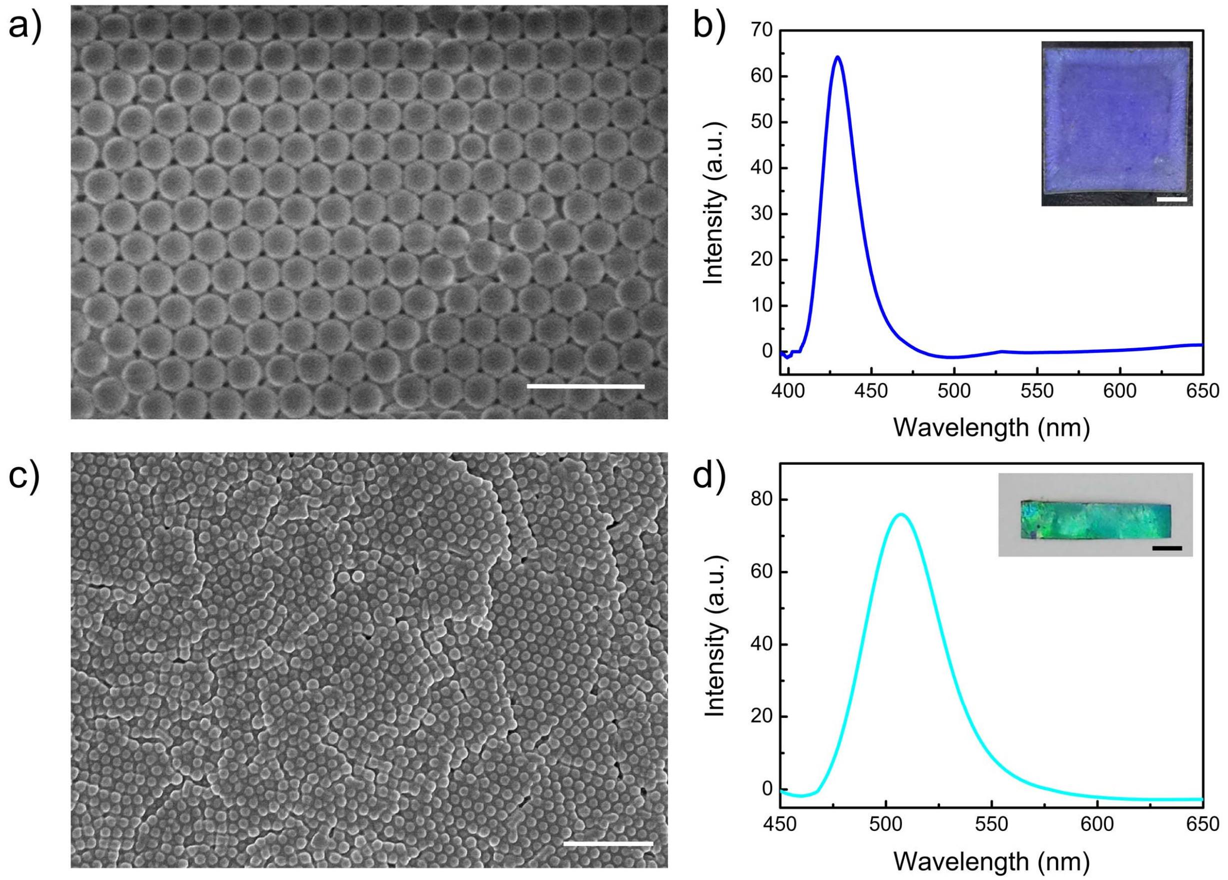

Figure S2. (**a**) SEM image of crosslinked PS nanospheres with diameter ~ 180 nm. Scale bar: 500 nm. (**b**) Reflection spectrum (ϑ = 90°) and photograph (inset) of the self-assembled photonic crystal film. Scale bar: 1 cm. (**c**) SEM image of crosslinked PS nanospheres embedded in PDMS matrix. Scale bar: 2 µm. (**d**) Reflection spectrum (ϑ = 90°) and photograph (inset) of the elastomeric photonic crystal film. Scale bar: 5 mm.

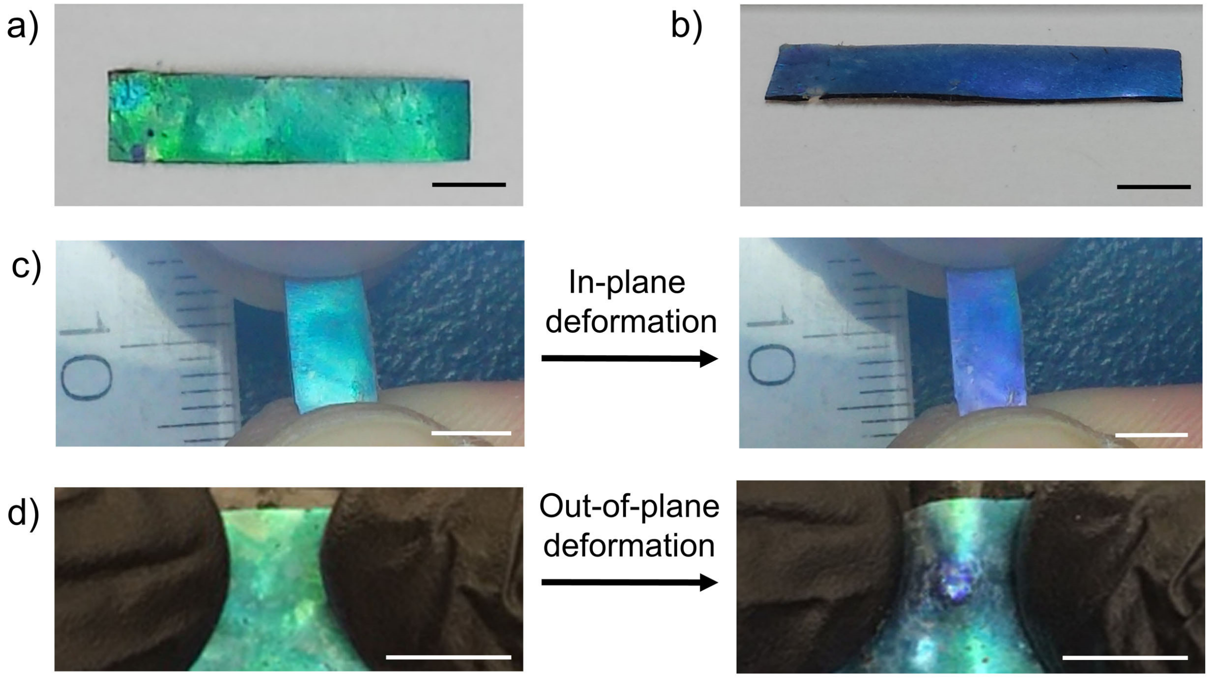

Figure S3. Photographs of (**a**) top view and (**b**) side view of the elastomeric photonic crystal film. (**c**) Photographs of the elastomeric photonic crystal film before and after being stretched. Scale bar: 5 mm. (**d**) Photographs of the elastomeric photonic crystal film before and after being deformed with a wooden stick. Scale bar: 1 cm.

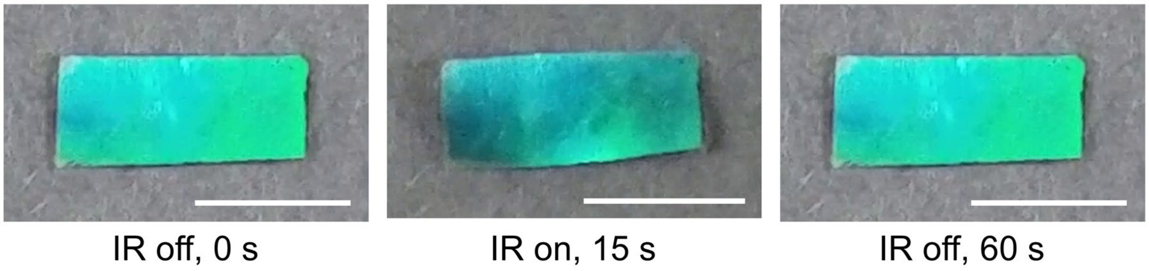

Figure S4. Photographs (top view) of reversible twisting and untwisting of the laminated film in response to global IR irradiation. The top-view photographs are taken at the fixed angle that is perpendicular to the substrate. Scale bar: 5 mm.

Table S1. Mechanical properties of the 0.1 wt% SWNT-LCE and laminated films at room temperature.

Materials	Tensile modulus (MPa)	Tensile strength (MPa)	Elongation at break (%)
0.1 wt% SWNT-LCE film	0.68 ± 0.04	5.5 ± 1.0	41.0 ± 4.9
Laminated film	0.70 ± 0.09	6.3 ± 1.3	57.3 ± 4.9

Supplementary Movie S1: Reversible bending actuation of the laminated film upon global IR irradiation.

Supplementary Movie S2: Inchworm-like walking of the laminated film on glass in response to on and off cycles of global IR irradiation.
